# Supplementary material for: The Effect of Vaccination with Live Attenuated Neethling Lumpy Skin Disease Vaccine on Milk Production and Mortality—An Analysis of 77 Dairy Farms in Israel
Source: Vaccines (Basel). 2020 Jun 19;8(2):324. doi: 10.3390/vaccines8020324 (PMC7350216; doi:10.3390/vaccines8020324)
Supplement: Supplementary file 1 [file vaccines-08-00324-s001.pdf]

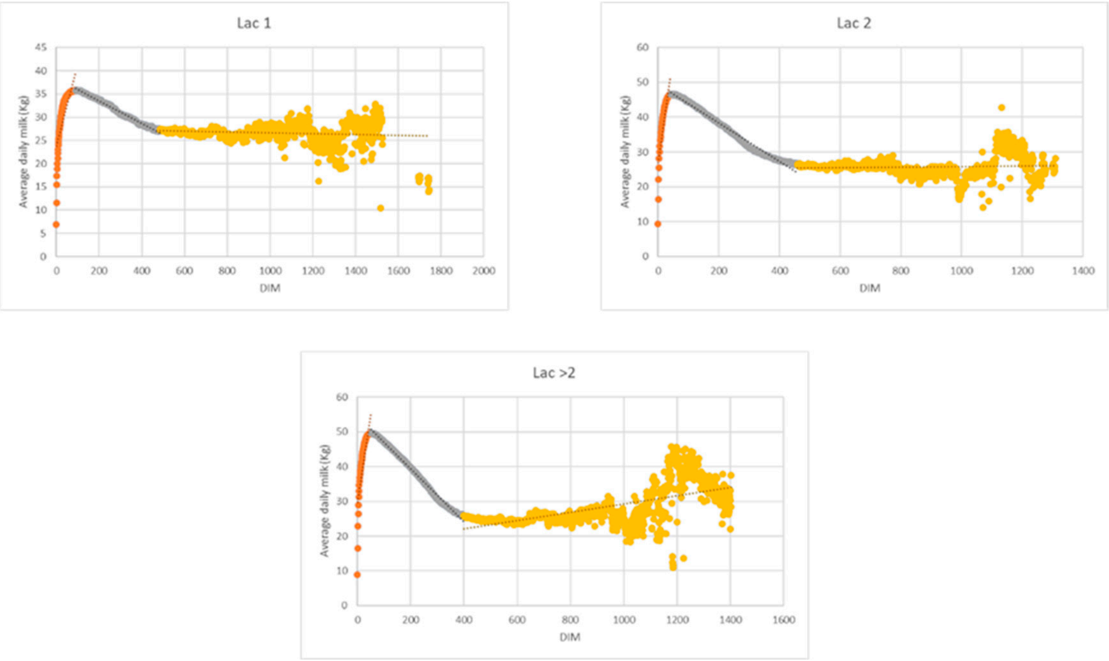

Figure S1. The average daily milk per DIM of 21,844 cows in 77 dairy cattle farms in Israel for lactation group 1 (Lac 1), lactation group 2 (Lac 2), lactation group >2 (Lac > 2). Three stages for each lactation group: Lac 1: 1. 0≤DIM≤91 2. 92≤DIM≤484 3. DIM≥485
Lac 2: 1. 0≤DIM≤41 2. 42≤DIM≤458 3. DIM≥459
Lac >2: 1. 0≤DIM≤50 2. 51≤DIM≤399 3. DIM≥400.
